# Model-Based Estimation of the Strength of Laser-Based Plastic-Metal Joints Using Finite Element Microstructure Models and Regression Models

**DOI:** 10.3390/ma14175004

**Published:** 2021-09-01

**Authors:** Julius Moritz Berges, Kira van der Straeten, Georg Jacobs, Jörg Berroth, Arnold Gillner

**Affiliations:** 1 Institute for Machine Elements and Systems Engineering, RWTH Aachen University, Eilfschornsteinstr. 18, 52062 Aachen, Germany; georg.jacobs@imse.rwth-aachen.de (G.J.); joerg.berroth@imse.rwth-aachen.de (J.B.); 2 Fraunhofer Institute for Laser Technology ILT, Steinbachstr. 15, 52074 Aachen, Germany; kira.van.der.straeten@ilt.fraunhofer.de (K.v.d.S.); arnold.gillner@ilt.fraunhofer.de (A.G.)

**Keywords:** hybrid joints, simulation, laser manufacturing, model-based engineering, microstructure model, mechanical design, surrogate modelling, product development

## Abstract

Plastic-metal joints with a laser-structured metal surface have a high potential to reduce cost and weight compared to conventional joining technologies. However, their application is currently inhibited due to the absence of simulation methods and models for mechanical design. Thus, this paper presents a model-based approach for the strength estimation of laser-based plastic-metal joints. The approach aims to provide a methodology for the efficient creation of surrogate models, which can capture the influence of the microstructure parameters on the joint strength. A parametrization rule for the shape of the microstructure is developed using microsection analysis. Then, a parameterized finite element (FE) model of the joining zone on micro level is developed. Different statistical plans and model fits are tested, and the predicted strength of the FE model and the surrogate models are compared against experiments for different microstructure geometries. The joint strength is predicted by the FE model with a 3.7% error. Surrogate modelling using half-factorial experimental design and linear regression shows the best accuracy (6.2% error). This surrogate model can be efficiently created as only 16 samples are required. Furthermore, the surrogate model is provided as an equation, offering the designer a convenient tool to estimate parameter sensitivities.

## 1. Introduction

Multi-material design is highly suitable to achieve lightweight goals and enable resource-efficient structures, especially in the automotive sector [1,2]. By using the right material in the right place, structures that are optimized to meet their specific requirements can be easily realized. One major multi-material approach is combining plastics and metals. Plastic-metal hybrids, however, require joining technologies that are suitable to combine the dissimilar materials with quite different material properties [3]. One promising joining technology for plastic and metal is thermal direct joining using a laser-structured metal surface. Laser-based thermal direct joining technology offers advantages over conventional joining technologies, as a high strength can be achieved, without having a complex process chain, as with adhesives, or requiring additional elements, such as bolts [4,5,6]. The cycle time, costs, and weight can be reduced compared to conventional joining processes. Thus, this joining technology is well suited for the transport sector, especially the automotive industry.

In the first step of the thermal direct joining process, a single-mode fiber laser is used to create linear grooves with undercut geometry on the metal surface. For this purpose, the laser beam is deflected over the metal surface at high scanning speeds, realized by a galvo scanning system, which enables the scanning of various structure patterns. Due to the high intensity of the laser beam, the metal is partially melted and sublimated, and the evaporation pressure ejects the surrounding melt out of the cavity, where it partially recasts on the edges. By repeating this process several times, an undercut is formed.

In the subsequent joining process, the plastic (thermoplastics) is joined by melting into the cavities, which results in mechanical interlocking and, depending on the material combination, specific adhesion [7]. The joining process can be carried out in different ways. On the one hand, plastic parts can be directly bonded by thermal direct joining, or, on the other, microstructured metal parts are integrated and bonded in a molding process (e.g., injection molding) [8].

**Figure 1:**
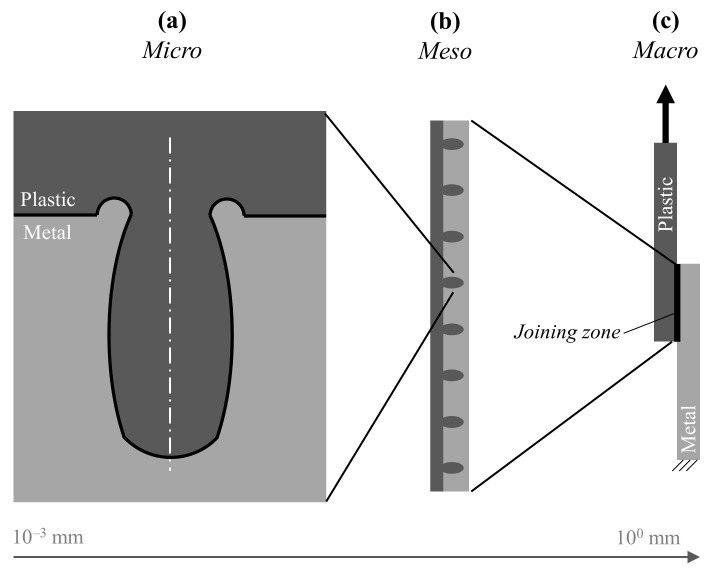
Characteristic levels for laser-based plastic-metal hybrid joints. Micro level (**a**) describes the properties of a single cavity. Meso level (**b**) describes the properties of multiple cavities, and macro level (**c**) describes the properties of the joining zone.

Depending on the shape of the cavity (Figure 1a) and the arrangement and orientation of the grooves in the joining zone (e.g., distance between cavities Figure 1b), the strength of the joint varies [9,10]. Usually, a smaller distance between the cavities and larger undercut result in higher joint strength [7,9]. The geometry of the microstructure can be adapted by different laser settings [7,11] and varies slightly for different metals. Previous investigations [6,7,10,11] show a variation in strength for different microstructures up to 25 MPa. 

Considering parameters from both micro and meso levels is essential for the mechanical design of laser-based plastic-metal joints [12]. Currently, the design is mainly based on experiments, which have to be repeated when changing the microstructure or the material. Changes in requirements and parameters (e.g., distance between cavities), as well as iterations, often arise in the product development process as requirements are further specified and tested [13]. To test these changes, to avoid expensive prototypes, and experiments as well as to perform optimizations in terms of interactions with other domains, besides computational costly higher-order models, reduced models of the mechanical behavior are required [14,15,16,17]. 

Although there is much research being conducted on the joining technology, there is insufficient work available on modeling the mechanical properties. Despite the high relevance of the plastic-metal interface [12], there are currently no experimentally validated physical models, such as finite element models, that can capture the influence of the microstructure on the strength. Furthermore, the application of surrogate models for the strength determination of laser-based plastic–metal joints has not yet been explored. 

The application of laser-based plastic-metal joints is inhibited due to the absence of appropriate design methods and efficient mechanical models, which can capture the effects on micro and meso levels and are suitable for product development. 

Therefore, the research questions that are addressed in this study are:

- Are simulation models suitable to capture the influence of microstructure parameters on the strength to consider them in the mechanical design?
- Are simplified surrogate models suitable to efficiently predict the strength of laser-based plastic metal joints?

The approach to answering these questions is structured as follows: In Section 2.1, the microstructure is parameterized based on microsections. A fully parameterized numerical finite element model of the plastic-metal joint is developed. In Section 2.2 statistical experimental design and surrogate modelling techniques are discussed. In Section 3, a use case is presented to demonstrate the approach by comparing the predicted strength of the experiment with the prediction of the numerical model and the surrogate models. From this, a methodology on how to create surrogate models for the mechanical design of laser-based plastic-metal hybrid joints is derived.

## 2. Modeling Methodology

### 2.1. Micro Model 

In the literature, besides modelling the production properties [18,19], only a few modelling approaches can be observed for the determination of the strength of laser-based plastic-metal hybrid joints. A comprehensive overview of modeling techniques of hybrid metal-composite interfaces can be found in [12].

In Ref. [20], an approach is presented to determine the homogenized mechanical properties of a single cavity using a finite element representative volume element (RVE) model. Engelmann et al. investigate the change in volumetric stretching for a linear elastic material behavior for 5 and 10 microstructures for two specific highly simplified cavity shapes, a smaller and a larger [7]. In [21], experimental and simulative investigations are conducted on laser-roughened PLA–aluminum joints. In an analytical and a numerical model, using a homogenized traction-separation interface, the effect of different production properties on the strength is analyzed. However, the roughened metal surface has no well-defined cavities and no distinct undercuts. 

These approaches do not consider fracture and contact behavior as well as the influence of individual microstructure parameters on the strength. Thus far, no numerical model or surrogate model has been presented and experimentally validated. Thus, in the following, a modelling approach for parametrization of the microstructure and an FE model which considers the defined parameters is presented. Based on the FE model, surrogate models are created and experimentally validated.

The geometric microstructural parameters are derived from several microsections. Only linear structures are considered, where the cavities are aligned linearly with equal distance on the joining zone. Therefore, the parametrization is reduced to a two-dimensional problem.

**Figure 2:**
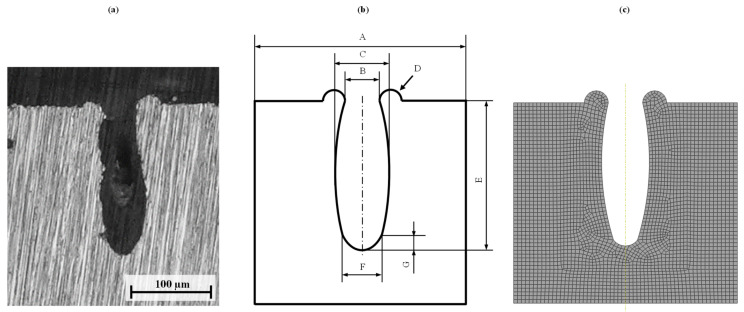
Microsection (**a**); parametrization rule (**b**); and FE model (**c**).

The parametrization rule shall be simple, and therefore require a minimum of parameters and not use any free-form surfaces. At the same time, the parametrization must be able to distinguish different types of shapes precisely to still capture individual influences in the microstructure. Therefore, a parameterization based on the shape of the cavity presented in Figure 2a via simple geometric shape elements (circles, arcs, etc.) is proposed as shown in Figure 2b. To simplify the parametrization rule and to enable its scalability, the parameters, except for the distance, are related to the parameter upper opening distance (parameter B, see Figure 2b).

Applying our parameterization rule, seven parameters are required to fully describe a microstructure in a cross-section (see Table 1 for a description of the parameters). Microscope investigations show that the microstructure is non-uniform, and the parameters vary with each individual cavity. Thus, the calculation of mean values from a set of at least five cavities is proposed.

A fully parameterized 2D finite element model (explicit) of the joining zone is developed. The model is executed via a Python API, which enables the automatic setup of joining zones with multiple cavities. Partitions are created for a finer mesh resolution in the areas around the cavities. Nearby the cavities, a quad-dominated meshing with a size of 0.01 mm is applied. Outside, a structured mesh with a size of 0.03 mm is used. The mesh sizes are determined by performing a mesh convergence study. For both plastic and metal parts, four-node plane stress elements are used. The normal interaction contact behavior between plastic and metal is defined as "hard contact" with surface-to-surface discretization. As plastic is usually the softer material and has a finer mesh, it is defined as the slave surface [22].

As the studies focus on non-polar plastics such as polypropylenes (see Section 3), specific adhesion in the interface between plastic and metal is not applied. The tangential behavior is defined using constant friction of µ = 0.2 [23]. From preliminary tests, it is determined that the polymer is failing in the interface and the metal neither breaks nor deforms drastically. Therefore, a linear elastic material behavior is applied for the metal part. Investigations by [24] show that, if short glass fiber reinforced plastics are used, the fibers are mostly chaotically distributed and oriented in the part and around the cavities. Thus, for the plastic part, plastic isotropic material behavior and a ductile damage model is applied.

### 2.2. Surrogate Modelling 

During product development, iterations frequently take place due to changes in parameters or requirements, resulting in a constant need for re-testing their fulfilment [25,26]. As previously described, the microstructure geometry changes for different settings of the laser or different materials and can therefore change in the product development stage. Numerical simulation models as presented in Section 2.1 have high accuracy, but they are not suitable to provide rapid feedback concerning parameter changes, as well as to perform optimization tasks, due to their high computation time. Thus, for model-based product development of laser-based plastic-metal joints, simplified surrogate models are required that represent the system’s behavior sufficiently accurately, but can be computed efficiently [16,27,28,29]. Furthermore, it is crucial for the design engineer that sensitivities of parameter changes can be quickly estimated and quantified.

**Figure 3:**
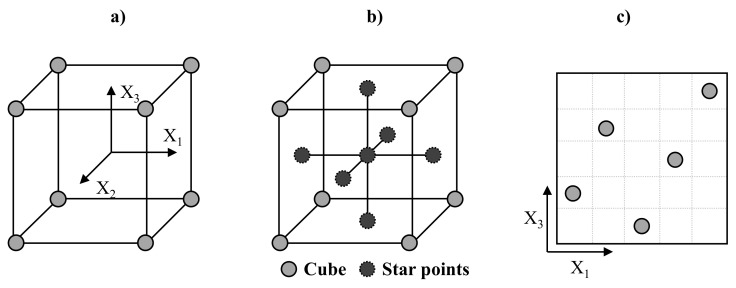
Full factorial plan (**a**) and face centered composite design (**b**) for three factors. Latin hypercube sampling (**c**) for two factors.

There are several different options for the calculation of surrogate models. An overview can be found in [30]. Each option requires both the generation of suitable samples and the selection and calculation of a suitable model fit [27]. A common approach for generating samples is statistical experimental plans (see Figure 3). The most common experimental design is a full factorial 2^k^ experimental design in which each parameter is tested at two levels (minimum and maximum) as shown in Figure 3a. This can be used to determine the main effects and interactions between parameters. To test second-order effects, central composite design plans can be used. Here, the full factorial design is extended by so-called star points. The position of the star points is defined by the value α. For α = 1, the star points lie on the faces of the cube (face centered central composite CCF) and each parameter is evaluated at three levels (Figure 3b).

In computer experiments, stochastic random methods such as Monte Carlo simulation or Latin hypercubes are widely used to generate samples. Latin hypercube [31] sampling (LHS) aims to increase the efficiency of Monte Carlo sampling. In LHS, each factor is divided into intervals. The individual samples are distributed in a way that there is exactly one data point on each axis (see Figure 3c). However, both MC and LHS are prone to gaps when few samples are available [32].

There are several ways of model fitting. Artificial neural networks (ANN) are becoming more and more popular in engineering design. However, ANN are mainly suitable for a large number of parameters and samples (~10.000) and are computationally expensive to create and train [27].

Polynomial regression is a widely used method for determining surrogate models [27]. Polynomial or linear regression is especially suitable for low-order problems. Furthermore, polynomial regression models show good results even with few samples, and can be easily implemented in product development [33]. As the result is a highly transparent equation, the model can be used well in practice by design engineers. In addition, sensitivities can be easily estimated based on the equation [34].

In model-based product development, model-related costs, i.e., building a surrogate model, the verification, and choosing the appropriate level of abstraction, have a major impact on the required effort and cost of the approach [35,36]. As the number of samples increases, the quality of the surrogate models improves [37]. However, due to the computational effort required, it is not feasible to generate an unlimited number of samples, so the minimum possible number of samples should be used [38].

Hence, a methodology for the generation of surrogate models for the mechanical design of laser-based plastic metal joints is required. The methodology needs to be reproducible, as simple as possible, and sufficiently accurate while requiring few samples to keep the effort low. 

Therefore, in the following, we want to explore which methodology (data point generation + model fit) is most suitable for this type of problem. A use case is applied in Section 3 and the estimated strength of the surrogate models is compared with the strength obtained from experiments and the numerical model. Finally, a methodology for creating new surrogate models for laser-based plastic–metal hybrids is proposed.

## 3. Demonstration of the Approach

### 3.1. Experimental Setup

#### 3.1.1. Materials and Sample Geometry

The metal used for the hybrid joint is regular stainless steel (1.4301, 1 mm thickness). During the joining process, the stainless steel is combined with a glass fiber reinforced Polypropylene. The GFRP used is X111F40-4/1-0/90° (Quadrant Plastic Composites AG, Lenzburg, Switzerland), which is a chopped fiberglass mat reinforced PP laminate with randomly oriented glass fibers and additionally reinforced fabric inside. The material thickness is 3 mm with a glass fiber content of 40 vol.-% (PP/GF40). The sample geometry is lap shear samples (100 × 25 mm^2^) with an overlap of 12.5 mm. The lap shear tests were performed according to DIN EN 1465 with a Zwick Z100 multi-purpose testing machine.

#### 3.1.2. Experimental Setup

The laser source used for the laser micro structuring process is a 1000 W continuous-wave (cw) single-mode fiber laser (IPG Photonics YLS-1000-SM), operating at 1064 nm wavelength. The laser beam is guided through an optical fiber into an Instelliscan25 scanning head (Scanlab), which deflects the laser beam on the metal surface. The metal is positioned in a device within the scan field to ensure reproducible patterns on the metal surface. A cross-jet with pressured air prevents the optics from contamination by process emissions. The focusing F-theta optics has a focal length of 330 mm creating a focal diameter of ~40 μm. The process parameters are influencing the geometry of the structure. A suitable parameter set, ensuring a reproducible shape of the cavities, is identified based on preliminary trials. The parameters used are:

- Laser power P = 750 W;
- Scanning speed v = 10 m/s;
- Structure distance 300 µm;
- Number of repetitions n = 2–7 (varied).

For the joining process, another laser system is used which consists of a cw diode laser (Laserline GmbH LDM 3000-100) which is mounted on a MOTOMAN-HP20-6 robot motion system. The maximum output power is 3000 W, and the laser system is operating at 900–1070 nm wavelength. A zoom optics (Laserline GmbH) with a focal length of 250 mm forms a square laser spot with a variable size from 5 × 5 mm^2^ up to 16 × 30 mm^2^. The variable spot size enables simultaneous irradiation of the entire joining area with a single pulse. In order to apply pressure and fix the sample arrangement a pneumatic clamping device with a sample holding fixture is used. A pressure of 1 bar is applied. The parameter set is determined on the basis of preliminary tests and ensures that the structures are properly filled with plastic:

- Laser power P = 660 W;
- Irradiation time t = 2.2 s;
- Joining area 25 × 12.5 mm^2^.

#### 3.1.3. Simulation Setup

**Figure 4:**
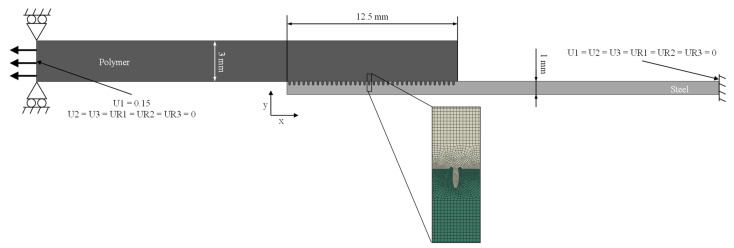
Setup of the FE Model (dimensions and boundary conditions) of the single lap joint.

The single-lap joint specimen is modelled by a finite element (FE) approach (see Figure 4). The microstructure parameters are derived from the test plans (see next Section). A fixed boundary condition is applied to the metal side, and a displacement boundary condition in the X-direction of 0.15 mm is applied to the plastic side, with rotation and displacement in the Y-direction being restricted. 

Material properties used for the steel and the plastic can be taken from Table 2. As described previously, a linear elastic material model is applied to the metal part and plastic isotropic material behavior and ductile damage model is applied to the plastic part, which degrades the stiffness of the material after damage initiation. Thus, the fracture behavior of the joint can be analyzed, and finally the strength of the joint can be determined. The reaction force RF1 in X-direction at the polymer surface is used as the target value. 

### 3.2. Microsection Analysis of the Test Specimens

**Figure 5:**
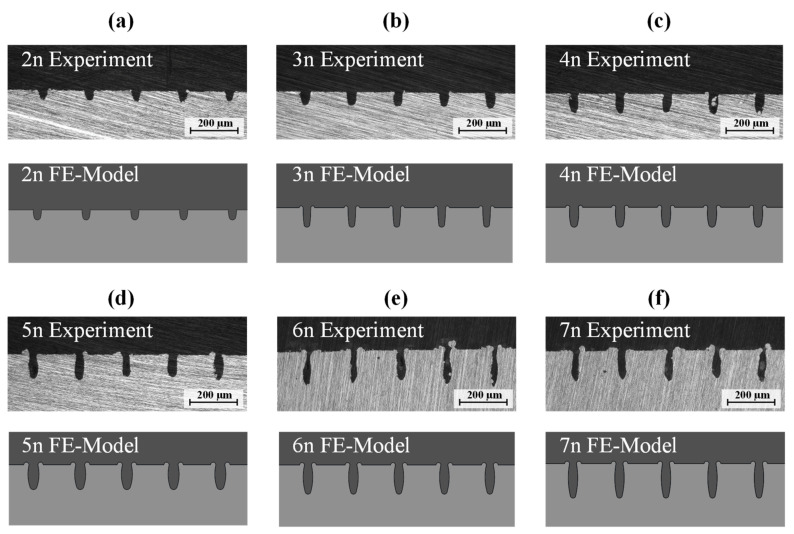
Comparison of microsections and FE-model for 2–7 number of runs are shown in (**a**–**f**).

Microsection analysis of the joining zone is performed for each specimen. The microsections and corresponding FE models are shown in Figure 5.

The parameters (see Table 3) of each cavity geometry are derived from the microsections according to the previously presented parameterization rule (see Figure 2b and Table 1). Furthermore, the maximum strength from the tensile shear tests is also given in Table 3. The maximum strength is calculated by the maximum load divided by the joining area (12.5 mm × 25 mm). The parameters Distance and Upper Opening are given as absolute values. The other parameters are defined as ratios to the Upper Opening (see Table 4). As there is no significant change in the bottom geometry parameters, i.e., lower arc length or lower arc height, these values are kept constant. Thus, five continuous parameters are investigated. 

For the sample generation and the calculation of surrogate models, lower and upper limits must be determined for each parameter. The models are intended to be valid within a range that can be realized in terms of laser production. Thus, minimum and maximum values for the parameters are derived based on the respective maximum and minimum values which are observed in the microsections (see Table 4).

The range for the distance is set to 250 µm–350 µm. Smaller distances are not reasonable due to the thermal influence during laser production which leads to poor undercuts and lower strength [7,39]. The larger the distance, the lower the strength of the joint. In order to take a practical and realistic value, the upper limit is set to 350 µm [7].

### 3.3. Surrogate Models

The methods studied for data generation are a face centered central composite design (CCF), full factorial and a half and quarter factorial. Additionally, LHS with the same sample size as the CCF design plan (43 samples) is used to test if a space-filling distribution performs better. To test whether a larger number of samples leads to better results, we carry out additional LHS with 200 data points. Table 5 gives an overview of the methodologies that are examined. 

MiniTab 2019 statistical and analysis software is used to create the statistical designs plans and model fits, as well as to analyze them. The python package pyDoE is used to create the LHS [40].

Regression models with the method of least squares are used as model fits. Initially, linear, interaction, and second-order model terms are considered. Then, the terms are reduced with backward elimination so that only significant terms remain in the model and the models are as compact as possible [41].

In addition, the application of neural networks as a model fit is examined. For ANN, a range of 2–10 hidden layers using a two-layer feed-forward network (Levenberg–Marquardt backpropagation) and different ratios of training to validation data is tested. However, pre-studies show that no useful model fits could be created with ANN. We conclude that the number of samples is not sufficient to train ANN for this type of problem.

## 4. Results and Discussion

The following section presents the results of the investigations and validation of the models by comparing the predicted strength of the surrogate models and the FE micro model with the strength from the experiments. To do this, the maximum load is calculated for each microstructure and its associated parameters (see Table 3) using the FE micro model and the different surrogate models.

**Figure 6:**
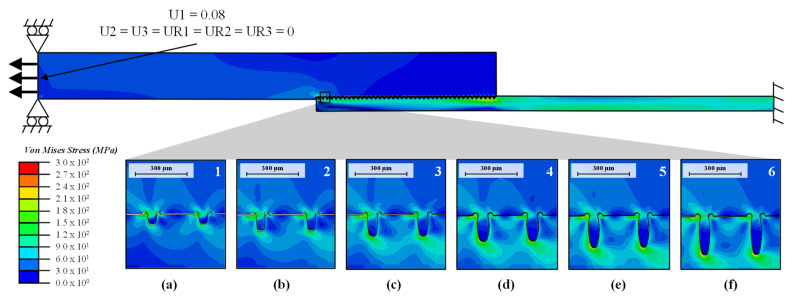
Illustration of the stress distribution (U1 = 0.08) of the single lap joint specimen of the finite element model. In (**a**–**f**) detailed sections for the different microstructures are shown.

Figure 6 shows the stress distribution in the specimen for a displacement of U1 = 0.08 mm obtained from the FE micro model. Larger details are also shown for the six different microstructures given in Table 3 for the most stressed cavities. In Figure 6a,b it can be observed that the plastic is pulled out of the cavity due to the lack of undercut. With increasing undercut (Figure 6c–f), the plastic is more clamped in the cavity. This phenomenon is a possible reason for the significant differences in the maximum strength of the various microstructures. 

**Figure 7:**
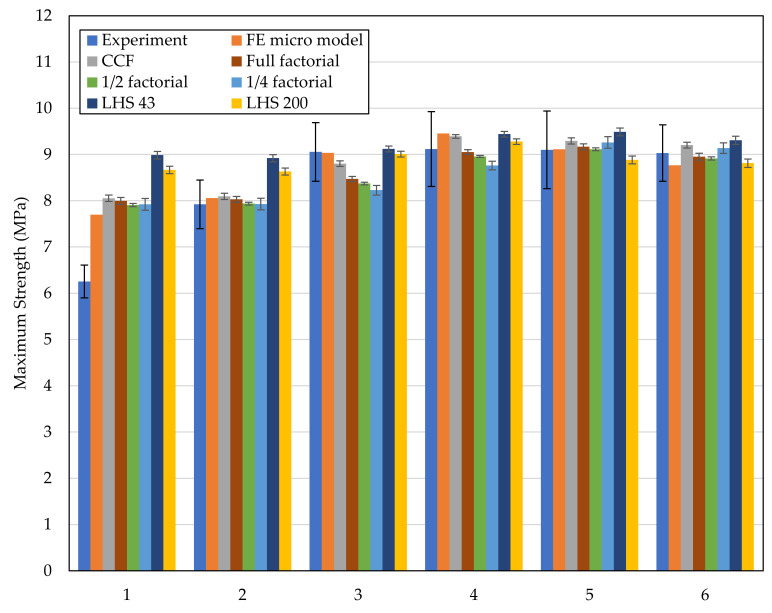
Validation of the approach by comparing the prediction of the strength of the surrogate models, FE micro model, and experiments.

Figure 7 shows the predicted strength of the joint based on the experiments, the FE micro model, and the surrogate models for the all the studied microstructures. In order to measure and compare the overall prediction accuracy of the models, the mean error of the models compared to the experiments and the coefficient of determination (R^2^) is calculated and shown in Table 5.

As expected, the FE micro model shows the best prediction accuracy of the strength with an error of 3.7% compared to the experiments. The surrogate models show a lower accuracy than the numerical micro model. The surrogate models based on statistical plans (factorial plans and central composite design) show similar small errors (~6–7%) and decent model fits (R^2^ > 90%). The surrogate model using the half-factorial plan shows the smallest error of 6.2% and the highest R^2^, which indicates a good model fit. Increasing the sample size from 8 to 16 results in a lower error. Further increasing the sample size to 32 (full factorial) or 43 (CCF) does not lead to a significant improvement of the prediction accuracy.

Using random sampling (Latin hypercube sampling LHS) with both 43 and 200 samples does not improve the prediction accuracy. The more uniform distributions do not provide advantages over the location of data points on the edges. Thus, for LHS, the number of samples needs to be substantially higher.

Using a half-factorial plan and linear regression model is recommended as the effort for creating it is low with only 16 samples required (for 5 factors). Furthermore, the model shows a high accuracy. The respective regression model is shown in Equation (1). The backwards elimination removes all non-significant effects and interactions. The physical behavior of the joint zone can be described by a compact equation. The sensitivities of each factor can be easily identified from this equation and used to derive guidelines for the design of laser-based plastic–metal joints. The studies show that a small Distance (A) and Aspect Ratio (E/B) have a positive effect on the strength. Large values for Upper Opening (B), Open Factor (C/B), and Bump Ratio (D/B) also show a positive effect on strength. By following these general guidelines, one can realize the best joints regarding the strength. These model-based results match previous experimental findings (cf. Section 1).

(1) $$\sigma_{\max,\;\mathrm{half}-\mathrm{factorial}}=1.25\;-\;0.0036\;\times A+0.022\;\times \;B\;-\;0.32\times \frac{E}{B}+6.03\;\times \frac{C}{B}+1.8\;\times \frac{D}{B}-\;0.006\times A\times \frac{C}{B}-\;0.006\times A\times \frac{D}{B}-0.29\times \frac{E\times C}{B}$$

Both the FE micro model and all surrogate models show the largest error for two (2n) and three (3n) laser runs (see Figure 5 and Table 3). In these configurations, the structures have no undercut (Open Factor = 1). Therefore, the FE model and surrogate models should be preferably used for structures with an undercut, i.e., Open Factor > 1, only. For 2n and 3n, many of the structures are pulled out of the metal cavities. For 4n to 7n, the fracture can be mostly observed in the neck area of the structures. This behavior explains the higher maximum load that can be achieved for structures with a distinctive undercut.

## 5. Conclusions

The application of laser-based plastic-metal hybrids is currently inhibited due to an absence of appropriate design methods and models, which can be used in product development. Therefore, in this paper, a methodology to calculate surrogate models for the strength estimation is presented. For this purpose, a parameterization rule for the microstructure is proposed and a novel numerical finite element micro model is developed. Different options for sample generation and model fits are compared in order to derive a suitable methodology for creating surrogate models. The outcomes can be summarized as follows:

- The presented numerical finite element (FE) micro model is able to predict the strength of laser-based plastic-metal joints with a 3.7% error. Thus, the parametrization rule and the assumption with averaging of irregular structures, contact, and friction definition, as well as the material behavior, seem appropriate.
- Simplified surrogate models can capture the influences of the microstructure parameters on the strength of laser-based plastic-metal joints.
- Half factorial experimental design with regression model-fit using backwards elimination shows the best prediction accuracy with an error of 6.2%.
- This surrogate model shows sensitivities, is highly transparent, and performs fast calculations. Thus, it can be used for model-based product development.
- Latin hypercube sampling (LHS) and artificial neural networks (ANN) cannot be applied due to the insufficient number of samples.

The presented approach shows that the strength of laser-based plastic-metal joints can be determined based only on models. Both the finite element micro model and the surrogate model are suitable for the integration into a model-based product development, which can help to avoid costly prototypes in product development. Especially in highly cost-driven sectors with short development cycles such as the automotive industry, this joining technology can now be better exploited.

Future work is planned in the integration of further parameters (e.g., temperature dependent behavior and dynamic behavior) and microstructure types (e.g., angle structures). We plan to extend and fully generalize with material parameters of the surrogate model to design all kinds of microstructures and joining zones and to derive more general design guidelines for laser-based plastic metal joints. Based on the sensitivities, developments in the field of lasers can be derived to produce optimal microstructures for maximum strength (e.g., higher Open Factor).

## Figures and Tables

**Table 1:** Parameters of the microstructure and their description.

Parameter	Description
Distance (µm)	A
Upper Opening (µm)	B
Aspect Ratio	E/B
Open Factor	C/B
Bump Ratio	D/B
Lower Arc Length Ratio	F/B
Lower Arc Height Ratio	G/B

**Table 2:** Material properties of the metal and plastic component.

	Steel (1.4301)	Plastic (PP/GF40)
Modulus (MPa)	200,000	9200
Density (g/cm³)	7.9	1.2
Poisson ratio	0.3	0.35
Tensile strength (MPa)	-	185
Strain at break (%)	-	2.3

**Table 3:** Parameterization of experiments with single lap joint and experimental strength.

		Design Variables					
ID	Number of Runs	Distance (µm)	Upper Opening (µm)	Aspect Ratio	Open Factor	Bump Ratio	Maximum Strength (MPa)
1	2n	300	51	1.22	1.00	0.5	6.26 ± 0.35
2	3n	300	51.5	1.86	1.00	0.5	7.92 ± 0.53
3	4n	300	56	2.32	1.09	0.5	9.05 ± 0.63
4	5n	300	57	2.75	1.25	0.6	9.12 ± 0.81
5	6n	300	46	4.17	1.39	0.7	9.10 ± 0.84
6	7n	300	45	4.93	1.36	0.7	9.03 ± 0.61

**Table 4:** Factors and limits for the sample generation.

Factor	Min	Max
Distance (µm)	250 µm	350 µm
Upper Opening (µm)	45 µm	57 µm
Aspect Ratio	1.2	5.0
Open Factor	1.0	1.4
Bump Ratio	0.5	0.7
Lower Arc Length Ratio	0.7 = const 0.5 = const	
Lower Arc Height Ratio		

**Table 5:** Mean error of the regression models compared to the strength of experiments.

Method	Samples	Mean Error (%)	R^2^ (%)
FE micro model	-	3.7	-
¼ factorial	8	7.1	93.07
½ factorial	16	6.2	99.74
Full factorial	32	6.3	91.78
CCF	43	6.8	98.57
LHS	43	11.3	83.82
LHS	200	9.1	52.13
